# Dynamic changes of monocytes subsets predict major adverse cardiovascular events and left ventricular function after STEMI

**DOI:** 10.1038/s41598-022-26688-9

**Published:** 2023-01-02

**Authors:** Maxime Boidin, Gregory Y. H. Lip, Alena Shantsila, Dick Thijssen, Eduard Shantsila

**Affiliations:** 1 Liverpool Centre for Cardiovascular Science, University of Liverpool, Liverpool John Moores University and Liverpool Heart & Chest Hospital, Liverpool, UK; 2 School of Sport and Exercise Sciences, Liverpool John Moores University, Liverpool, UK; 3 Department of Clinical Medicine, Aalborg University, Aalborg, Denmark; 4 Department of Physiology, Research Institute for Health Sciences, Radboud University Medical Center, Nijmegen, The Netherlands; 5 Primary Care, University of Liverpool, Liverpool, UK; 6 Department of Sport and Exercise Sciences, Institute of Sport, Manchester Metropolitan University, Manchester, UK

**Keywords:** Atherosclerosis, Outcomes research

## Abstract

We explored how dynamic changes in monocyte subset counts (as opposed to static values to specific time points), and their phagocytic and NFκB activity relate to major adverse cardiovascular events (MACE) and left ventricular ejection fraction (LVEF) in patients with ST-elevation myocardial infarction (STEMI). Changes in counts, phagocytic activity and intracellular levels of inhibitory κB kinase β (IKKβ) (a marker of NFκB activity) of monocyte subsets (CD14^++^CD16^−^CCR2^+^ [Mon1], CD14^++^CD16^+^CCR2^+^ [Mon2] and CD14^+^CD16^++^CCR2^−^ [Mon3]) were measured by flow cytometry in patients with STEMI at baseline, and again after one week, two weeks, and one month. LVEF was measured by echocardiography at baseline and six months after STEMI. Baseline data included 245 patients (mean ± SD age 60 ± 12 years; 22% female), who were followed for a median of 46 (19–61) months. Multivariate Cox regression demonstrated that more prominent dynamic reduction in Mon2 by week 1 (n = 37) was independently associated with fewer MACE (HR 0.06, 95% CI 0.01–0.55, *p* = 0.01). Also, less prominent reduction in Mon2 at month 1 (n = 24) was independently predictive of 6-month LVEF. None of the other dynamic changes in monocyte subsets were associated with changes in survival from MACE. Neither phagocytic activity nor IKKβ were associated with survival for each monocyte subset. We showed how distinct pattern of dynamic changes in Mon2 are related to both MACE risk and recovery of cardiac contractility. Further research is needed to understand the mechanism of the monocyte effect and possibilities of their pharmacological manipulation.

## Introduction

The pathophysiology of myocardial infarction (MI) is characterized by vascular inflammation, plaque rupture, thrombosis and coronary artery occlusion, resulting in myocardial necrosis^1^. Monocytes and macrophages are essential components of innate and adaptive immunity and are implicated in many inflammatory diseases^2^, including atherosclerosis^3–5^, where monocytosis has been associated with impaired recovery and unfavourable prognosis following MI^6,7^. However, monocytes are also involved in angiogenesis and myocardial healing after MI^8,9^ via their phagocytic activity and the release of biologically active molecules. As a result, monocytes can influence left ventricular remodelling^10^, and play a significant role in cardiovascular health^11^.

Human blood monocytes include three subsets: classical CD14^++^CD16^–^ (Mon1), intermediate CD14^++^CD16^+^ (Mon2), and non-classical CD14^+^CD16^++^ (Mon3) monocytes^12,13^. These monocytes differ in their relative frequency, phenotype, and function. Mon1 represent the highest proportion of the monocytes (≈85%), while Mon2 and Mon3 represent about 5 and 10%, respectively^14^. Our group has demonstrated that high absolute Mon1 counts during acute MI were associated with more major adverse cardiovascular events (MACE)^15^ and worse myocardial salvage and convalescent left ventricular ejection fraction (LVEF)^16^. Higher baseline counts of Mon1 and Mon2 were positively associated with baseline and 6-month follow-up global longitudinal strain (GLS)^17^. Higher post-MI Mon2 counts were independently predictive of MACE and heart failure (HF). Also, higher intracellular levels of inhibitory κB kinase β (IKKβ), which is a cytoplasmic marker of activation of the nuclear factor-κB (NFκB) pathway, were associated with tenfold lower occurrence of HF^15^.

In addition to static levels of monocytes, also dynamic changes in these monocytes following MI may have potential predictive capacity. Mouse studies have shown distinct dynamic changes in monocyte subset counts and their different roles at different stages of recovery after MI. In mouse models, CCR2^hi^Ly6C^+^ monocytes (resemble human Mon1) were numerous in the myocardium during first four days after MI, but sparce later^9,18,19^. This contrasted to the opposite trend for CXCRI^hi^Ly6C^−^ monocytes (resemble human Mon3)^9,18,19^ CCR2^hi^Ly6C^+^ thus dominate in early phase and exhibits phagocytic activity, and inflammatory process, while CXCRI^hi^Ly6C^−^ dominate later, attenuates inflammatory properties, and expresses proangiogenic vascular-endothelial growth factor. Overall, CCR2^hi^Ly6C^+^ appear to be associated with tissue damage, while CXCRI^hi^Ly6C^−^ principally promotes healing by myofibroblast accumulation, angiogenesis, and deposition of collagen^9^. Our previous work has shown that, as with Mon1, Mon2 was more functionally active in the first few days after MI^20^. Mon2, which is hardly represented in mice has multiple unique phenotypic (*e.g.*, highest of all monocyte expression receptors to angiogenic factors) and functional (*e.g.*, highest phagocytic activity) properties, which open speculation about their role in post-MI recovery^21^.

Whilst most studies assessed predictive values of the subsets at fixed time points, their dynamic changes could be useful to investigate the balance between myocardial inflammation and healing after MI. In this study, we aimed to establish for the first time whether dynamic changes in monocyte subsets and their changes in phagocytic and NFκB activity post-ST-elevation MI (STEMI) are related to clinical outcomes and LVEF.

## Materials and methods

Study design and participant recruitment are detailed in the Online Resource 1. All patients undergone primary percutaneous coronary intervention (PCI)^22^. Measurements of flow cytometry, intracellular activation of nuclear factor κB (NFκB) pathway, and cardiac function are detailed in the Online Resource 1. Briefly, peripheral venous blood was collected after primary PCI within the first 24 h from admission (baseline) and several follow up time points. Monocyte subsets were quantified and characterized using flow cytometry within 60 min of blood sampling. Plasma was obtained by centrifugation and stored at − 70 °C for batched analyses. Cardiac function was assessed at 3 days and 6 months after STEMI. The study was performed in accordance with the Helsinki declaration and was approved by the Coventry Research Ethics Committee (approval number 09/H1210/11). All participants provided written informed consent.

Statistical analyses are detailed in the Online Resource 1. The dynamic changes in monocyte subsets were analysed as the difference in their characteristics between follow up time points and baseline. To allow sufficient power for statistical analyses, we only included time points with data from 40 or more patients available.

### Outcome events

The study outcome was the first occurrence of a MACE defined as recurrent acute coronary syndrome (unstable angina, or non-STEMI, or STEMI with the presence of 2/3 criteria: that is, typical chest pain, electrocardiographic ischemic change, or elevated troponin T)^23^, new clinical diagnosis of congestive HF based on symptoms and echocardiographic evidence of left ventricular dysfunction or death. The analysis excluded two cases of periprocedural death on the day of STEMI. Patients were followed up using electronic hospital records from each recruitment site. Patients who were not reviewed in hospital were contacted to enquire about any events that were not recorded by their local hospital.

## Results

We enrolled 245 patients admitted with STEMI (mean ± SD age 60 ± 12 years; 22% female). The study was done within the timeframe of the project ethical approvals. Longer outcome collection was not possible, and data were now fully anonymised. During follow up of a median of 46 (19–61) months, 82 (33%) patients developed a MACE. The MACE events included 35 (43%) HF, 33 (40%) recurrent acute coronary syndrome, and 14 (17%) deaths. Patients who developed MACE were older (mean difference [Δ] 7 years), had a higher post-MI troponin T level (Δ5,937 ng/L), and a lower eGFR (Δ9 mL/min/1.73 m^2^). Because of blood sampling logistics and ability of patients to attend follow up, blood samples were taken at 7.4 ± 0.9 days (week 1), 15.6 ± 3.1 days (week 2), and 32.0 ± 6.3 days (month 1) for the included time points. As expected, patients who developed MACE had a higher proportion of cardiovascular risk factors (Table 1).

**Table 1:** Clinical characteristics of the patients.

	All (n = 245)	No MACE (n = 163)	MACE (n = 82)	*p* value
**Baseline characteristics**				
Age, years	61 [52–68]	58 [50–66]	67 [57–73]	< 0.001
Males, n (%)	190 (78)	124 (76)	66 (80)	0.43
Body mass index, kg/m^2^	26.2 [25.2–28.8]	26.3 [25.3–29.4]	26.1 [24.8–27.7]	0.19
Troponin T, ng/L	2340 [1080–6020]	1958 [859–4630]	3930 [1692–9352]	0.001
eGFR, mL/min/1.73 m^2^	77 [63–90]	84 [65–90]	69 [55–85]	< 0.001
Total cholesterol, mmol/L	4.8 [4.4–5.2]	4.8 [4.3–5.2]	4.9 [4.5–5.3]	0.39
**Previous medical history**				
Hypertension, n (%)	125 (51)	74 (46)	51 (62)	0.01
Diabetes mellitus, n (%)	57 (23)	31 (19)	26 (32)	0.03
Myocardial infarction, n (%)	33 (14)	14 (9)	19 (23)	0.002
Percutaneous coronary intervention, n (%)	22 (9)	11 (7)	11 (13)	0.09
Coronary artery bypass grafting, n (%)	12 (5)	6 (4)	6 (7)	0.22
Cerebral vascular accidents, n (%)	18 (7)	8 (5)	10 (12)	0.04
Chronic obstructive pulmonary disease, n (%)	29 (12)	20 (12)	9 (11)	0.78
Smokers, n (%)	137 (56)	96 (59)	41 (50)	0.17
LVEF at day 3, %	51 [43–57]	55 [47–59]	42 [36–52]	< 0.001
LVEF at month 6, %*	50 [43–56]	52 [48–57]	42 [33–54]	< 0.001
LVEF change, %	− 2 [(− 8)− 6]	-3 [(-8)-5]	− 1 [(− 9)− 8]	0.72

Continuous variables are expressed as mean ± SD for normal data or median [IQR] for non-normal data; dichotomous variables are expressed as number and percentage.

*eGFR* estimated glomerular filtration rate, *IQR* interquartile range.

*p* values represent the unpaired *t*-test analyses between MACE and no MACE for normal data and Mann–Whitney non-parametric test for non-normal data (troponin T and tumour necrosis factor α).

*p < 0.05, time-effect month 6 *vs.* Day 3.

**Figure 1:**
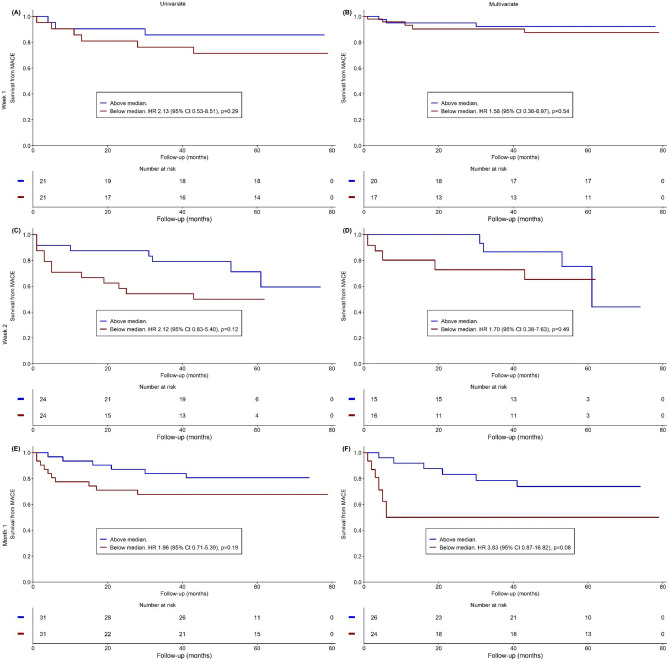
Predictive value of Mon1 for MACE. Univariate (left column) and multivariate (right column) **s**urvival analyses from MACE in Mon1 according the changes in blood counts of monocyte subsets at one week (**A**, **B**), two weeks (**C**, **D**), and one month (**E**, **F**). *MACE* Major adverse cardiovascular events, *HR* Hazard ratio, *CI* Confidence interval.

**Figure 2:**
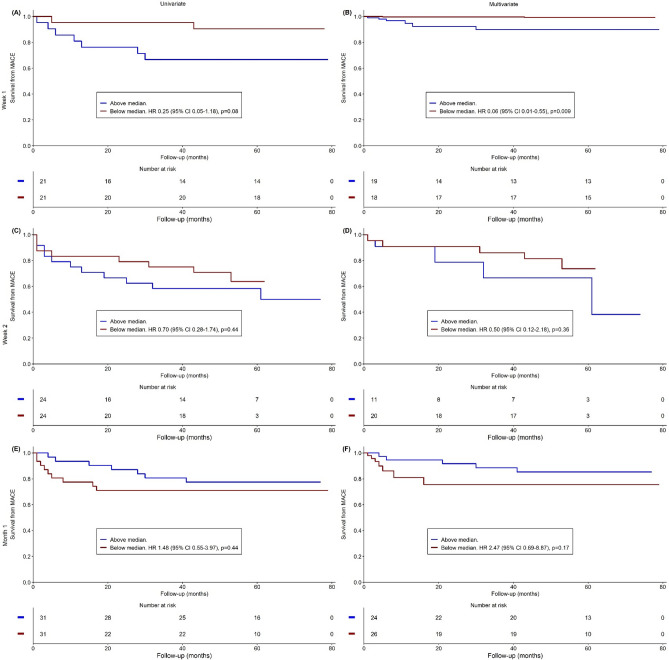
Predictive value of Mon2 for MACE. Univariate (left column) and multivariate (right column) **s**urvival analyses from MACE in Mon2 according the changes in blood counts of monocyte subsets at one week (**A**, **B**), two weeks (**C**, **D**), and one month (**E**, **F**). *MACE* Major adverse cardiovascular events, *HR* Hazard ratio, *CI* Confidence interval.

**Figure 3:**
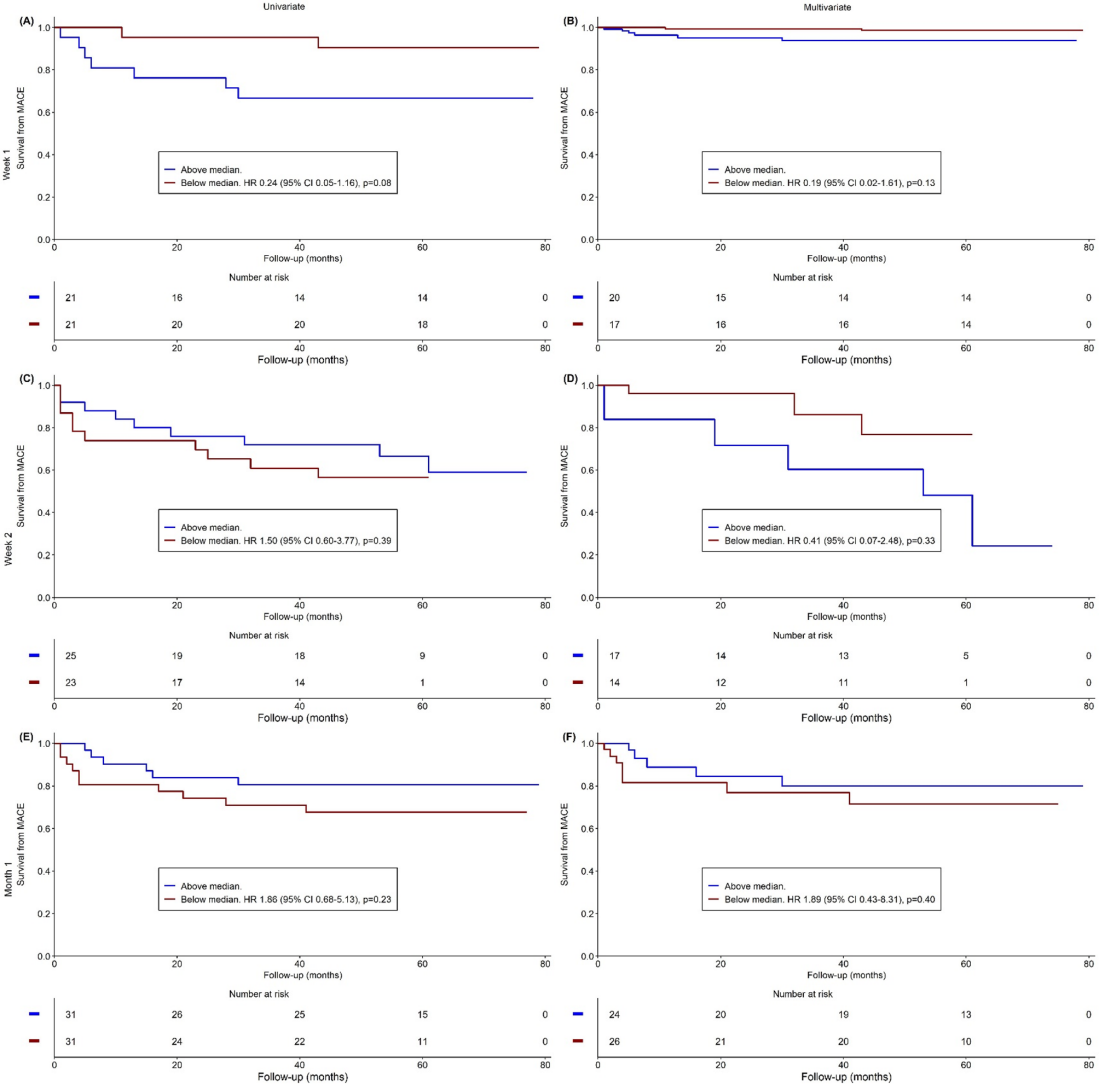
Predictive value of Mon3 for MACE. Univariate (left column) and multivariate (right column) **s**urvival analyses from MACE in Mon3 according the changes in blood counts of monocyte subsets at one week (**A**, **B**), two weeks (**C**, **D**), and one month (**E**, **F**). *MACE* Major adverse cardiovascular events, *HR* Hazard ratio, *CI* Confidence interval.

Data at week 2 and month 1 did not always come from the same group of patients than data at week 1. This makes MACE rates variable for each time point. Dynamic changes of monocyte subsets were analysed at week 1 (n = 42), week 2 (n = 48), and month 1 (n = 62) post-MI. The values of the dynamic changes in monocyte characteristics are presented in the Online Resource 2 and their predictive values for MACE is shown on Figs. 1, 2 and 3. More prominent reduction in Mon2 by week 1 tended to be associated with fewer MACE on univariate analysis (HR 0.25, 95% CI 0.05–1.18, *p* = 0.08) (Fig. 2A), and was significantly associated with fewer MACE on multivariate analysis (HR 0.06, 95% CI 0.01–0.55, *p* = 0.009) (Fig. 2B). More prominent reduction in Mon3 by week 1 tended to be associated with fewer MACE on univariate analysis (HR 0.24, 95% CI 0.05–1.16, *p* = 0.08) (Fig. 3A), but was not predictive of MACE on multivariate analysis (HR 0.19, 95% CI 0.02–1.61, *p* = 0.13) (Fig. 3B). The pace of change other tested dynamic monocyte changes also was not predictive of MACE.

Less prominent reduction in Mon2 counts by month 1 was associated better 6-month LVEF on univariate (*p* = 0.03) and multivariate analysis (*p* = 0.003) (Table 2). Dynamic changes of other subsets and Mon2 changes by week 2 were not related to 6-months LVEF (*p* > 0.05 for all). Week 1 changes and were not assessed due to insufficient number of samples (n = 4).

**Table 2:** Predictive value of changes of monocyte subsets for left ventricular ejection fraction at six months and changes in LVEF from baseline.

	Univariate			Multivariate		
	B ± SE	*β*	*p* value	B ± SE	*β*	*p* value
**LVEF at six months**						
*Changes at week 2 (n* = *35)*				*(n* = *24)*		
Mon1	0.003 ± 0.008	0.075	0.67	− 0.009 ± 0.010	− 0.231	0.41
Mon2	− 0.020 ± 0.017	− 0.205	0.24	− 0.012 ± 0.020	− 0.136	0.58
Mon3	− 0.077 ± 0.049	− 0.263	0.13	− 0.029 ± 0.092	− 0.097	0.75
*Changes at month 1 (n* = *31)*				*(n* = *24)*		
Mon1	0.011 ± 0.008	0.239	0.20	0.023 ± 0.013	0.498	0.10
Mon2	− 0.039 ± 0.017	− 0.397	**0.03**	− 0.084 ± 0.023	− 0.633	**0.003**
Mon3	− 0.03 ± 0.054	− 0.102	0.59	0.002 ± 0.093	0.006	0.98

B: Regression coefficient; SE: Standard error; LVEF: Left ventricular ejection fraction; *β*: adjusted regression coefficient.

Multivariate analyses were performed using age, sex, maximal troponin T levels, estimated glomerular filtration rate, history of diabetes and smoking and monocyte subset counts.

Significant values are in bold.

Due to small number of samples, we did not analyse predictive value of week 1 dynamics of monocyte phagocytic activity (n = 3). Predictive value of week 1 intracellular IKKβ were not analysed since they were not interpretable*.* The dynamic changes of the tested monocyte phagocytic activity and intracellular IKKβ levels were not predictive of MACE (See Online Resource 3).

## Discussion

To the best of our knowledge, our study represents the first cohort study of dynamic monocyte subsets changes after STEMI. First, greater decrease in Mon2 at week 1 was independently associated with a lower risk of MACE, whilst none of the other changes in monocyte subsets were associated with significant changes in survival from MACE at any of the time points. Second, less prominent reduction in Mon2 by month 1 was independently associated with better 6-month LVEF. Third, neither changes in phagocytic activity nor changes in IKKβ were associated with survival from MACE. Our data present novel evidence that dynamic changes in specific monocytes subsets, especially Mon2, are related to cardiac function and the survival from MACE.

Several studies revealed significant differences in static blood counts of monocyte subsets between healthy and cardiac individuals. For example, Berg et al.^24^ showed that Mon1 was elevated in 700 individuals who developed ischemic cardiovascular events over a 15-year follow-up, independent of gender, age, and cardiovascular risk factors compared to their counterparts. Similarly, individuals with acute MI demonstrated a 2.5-fold increase in Mon2 compared to healthy individuals^20^. Our study specifically focused on post-MI dynamic changes in monocytes. Interestingly, despite the overwhelming evidence that Mon1 is elevated following MI and has independent prognostic value for future (cardiovascular) events, we found no evidence that dynamic changes in Mon1 following MI have prognostic value. In contrast, a decrease in Mon2 in the early stages (*i.e.*, one week) was associated with significant change in survival from MACE in our study. Recently Mon2 increase during the first week after STEMI was linked to higher 2.5-year mortality^25^. Our study expands the evidence by showing that adequate Mon2 decrease is essential to reduce the risk of MACE. Mon2 in the early stages after MI (*i.e.*, day one) were correlated with peak troponin level and plasma cytokines^20^, demonstrating a close relationship between Mon2 characteristics and the degree of myocardial damage and recovery following STEMI. This is of special interest as post-MI levels of peak troponin are independently related to subsequent (cardiovascular) events. Moreover, this latter study also found that dynamic changes in Mon2 characteristics following MI are related to LVEF at 6 weeks. That is, decrease in Mon2 at day 1 was independently predictor of higher LVEF at 6 weeks (β = 0.37, *p* = 0.01). These dynamic effects of Mon2 agree well with recent data on higher Mon2 being related to larger post-MI myocardial scaring on cardiac magnetic resonance imaging^26^ being associated with excessive blood levels of vascular endothelial growth factor (VEGF) 6 h following coronary intervention after STEMI^27^. Finally, neutrophil extracellular traps (NETs), network structures of extracellular fibres implicated in immune-mediated disease, have been positively associated with infarct size^28^. At the same time, Mon2 are enriched in the region of the myocardial infarct damage. Taken together, this supports our observation that post-MI dynamic changes in Mon2 is related to LVEF at 6 weeks.

Although Mon2 levels prevail over Mon3 in the early stages following STEMI, Mon3 levels predominate at 30 days later in stable coronary artery disease^20^. However, we did not find any association between changes in Mon3 at one month and change in survival from MACE in our study. Mon3 shows a smaller phagocytic activity, a lower inflammatory activity, lower activity of IKKβ^21^, as well as a lower production of TNF-α, IL-6, and IL-1β in response to lipopolysaccharide (which stimulates immune response by interacting with the membrane receptor CD14), and a lower rate of aggregation with platelets^21,29^ compared to Mon1 and Mon2. Taken together, this could explain why changes in Mon3 was not associated with changes in survival, independently of age, sex, maximal troponin T levels and estimated glomerular filtration rate, and history of diabetes and smoking, while increase in Mon2 at week 1 has a positive impact on survival from MACE.

Our observations raise the question about the potential underlying mechanisms. Monocytes are short-lived circulating cells that are implicated in inflammation and/or healing through both direct effects and by differentiation into dendritic cells and macrophages. Moreover, under normal conditions, dendritic cells, which play a key role in T-cell activation, promote a tolerogenic environment through the expression of the immunosuppressive enzyme indoleamine 2,3-dioxygenase (IDO). Lipopolysaccharides (LPS) contribute to the inflammatory processes that lead to CAD and decrease Mon2 after MI^30^. In the context of STEMI, dendritic cell maturation is altered after stimulation with LPS (*i.e.*, a stimulus for T-cell activation), suggesting their role in T-cell dysregulation^31^, and in turn, inflammation and atherogenesis^32^. The specific pro-inflammatory Th17 T-cell is exaggerated in patients with STEMI compared to patients with stable angina or non-STEMI but is also reduced in patients who showed clinical improvement (*i.e.*, no recurrent cardiovascular events)^33^. Monocytes develop from the common myeloid progenitor in the bone marrow and are released into the circulation, where they comprise their subsets^2^. Mon2 seems to be also mobilized from spleen depot in mice rather than from bone marrow^19^, but the proportion between bone marrow and spleen in the circulation in individuals with STEMI remains unclear^19^. Monocytes and macrophages are implicated at the three stages post-MI. The initial phase includes a pro-inflammatory response, followed by a second phase where monocytes and macrophages return to baseline, while macrophages persist for months after MI for remodelling the myocardium (last phase)^9,34^. Interestingly, Mon1 and Mon3 are the only subsets associated with endothelial dysfunction^35^. However, even if endothelial dysfunction precedes development of atherosclerosis and further increases risk of cardiovascular events, our data indicated that this association did not lead to changes in survival from MACE. Our results for Mon2 are in concordance with another study where Mon2 independently predicts cardiovascular events in a cohort of 951 patients referred for elective coronary angiography^20^. Mon2 has a role in myocardial damage^20^. For example, coronary artery disease is associated with higher expression of interleukin (IL)-6, a cytokine present during the acute inflammatory phase, on Mon2^36^. This higher expression of IL-6 seems to be induced by an increase secretion of IL-17^37^, another inflammatory cytokine that is involved in the activation of leukocytes. Moreover, troponin T levels is correlated with changes in Mon2 in patients with acute MI, unstable angina, acute HF, or stroke^20^. Finally, Mon2 are associated dyslipidaemia, plaque vulnerability and rupture in patients with STEMI^38^. Taken together, this could partly explain why dynamic changes in Mon2 is associated with changes in survival from MACE in patients post-MI.

Since phagocytosis is a central function of monocytes, and especially relevant in relation to MI, we also examined changes in post-MI phagocytic activity of each monocyte. Nonetheless, our study demonstrated that changes in IKKβ, and changes in phagocytic activity of each monocyte were not associated with survival from MACE. Compared to Mon3, Mon2 may possess a higher pro-inflammatory profile, as in vitro studies found an enhanced production of ROS, TNF-α and interleukin-1 (IL-1)^39,40^. Thus, the association between dynamic changes in monocyte subsets and survival from MACE might be explained by a change in the inflammatory profile of Mon2. Even if IKKβ plays a role in monocyte recruitment and atherogenesis^41^, our study failed to demonstrate that changes in IKKβ of each monocyte subset were associated with survival from MACE. Taken together, we cannot confirm that it is the pro- or anti-inflammatory profile of the monocyte subsets that is responsible of the change in survival from MACE in patients with STEMI.

### Limitations

Some limitations are evident in this study. The main limitation relates to the sample size. Although we included a large baseline sample size (n = 245), for different availability or logistic reasons, blood counts of monocyte subsets post-MI were not available in all participants, leading to relatively small subsets available for the statistical analysis. Nonetheless, we found a robust effect of the dynamic change in Mon2 following MI in relation to subsequent survival and remodelling. Moreover, this study is the first to investigate dynamic changes in monocytes subsets in relation with survival from MACE, and clearly demonstrated that different changes occurred and led to different clinical outcomes. A further limitation relates to the timing of blood collection. We tried to collect blood samples as soon as possible following PCI, as variation of a few hours in blood sampling seems to affect monocyte characteristics. For this reason, we have considered the time between PCI and blood sampling as a random factor in our statistical analysis to effectively minimise its potential impact on our results. The fact that blood monocyte subsets were assessed in the circulation, whereas this blood count may not fully reflect the process and actions of these monocytes in the myocardium. This limitation is especially relevant for Mon3 which is demonstrated to be important for post-MI salvage in mice^9,19^. While the study focused on the counts of monocyte subsets, their functional status is likely equally important. The functional assessment was beyond the scope of this study, but consideration of the subset functions has been given but putting the findings in the context of other complementary research.

### Conclusions

We show how distinct pattern of dynamic changes in counts of monocyte subsets affect prognostication following STEMI, with especially changes in Mon2 being related to both MACE risk and recovery of cardiac function following MI. Further research is needed to understand the mechanism of the monocyte effect, its potential clinical relevance in predicting post-MI outcomes, and hence explore possibilities for (pharmacological) manipulation of these post-MI dynamic changes in monocytes to improve post-MI survival.

## Supplementary Information

Supplementary Information 1.

Supplementary Information 2.

Supplementary Information 3.

## Data Availability

The datasets generated during and/or analysed during the current study cannot be publicly shared as it has not been included in the ethics during the sample collection, but are available from the corresponding author on reasonable request.
