# Supplementary material for: Dynamic changes of monocytes subsets predict major adverse cardiovascular events and left ventricular function after STEMI
Source: Sci Rep. 2023 Jan 2;13:48. doi: 10.1038/s41598-022-26688-9 (PMC9807564; doi:10.1038/s41598-022-26688-9)
Supplement: Supplementary file 1 — Supplementary Information 1. [file 41598_2022_26688_MOESM1_ESM.docx]

**Online Resource 1**

**Materials and methods**

*Study design and participant recruitment*

The study included 245 patients admitted with STEMI to City Hospital (n=84), Sandwell General Hospital (n=32), Birmingham Heartlands Hospital (n=107) or Queen Elizabeth Hospital Birmingham (n=22) in Birmingham, United Kingdom between November 2009 and November 2012. STEMI was diagnosed according to the European Society of Cardiology criteria, all patients undergone primary percutaneous coronary intervention (PCI) [1]. Exclusion criteria were infectious diseases (*e.g.,* sepsis), inflammatory disorders (*e.g.*, rheumatoid arthritis, psoriasis, systemic lupus erythematosus) that required treatment with steroids and other immunosuppressive agents, active cancer, severe renal failure (*i.e.*, eGFR < 15 mL/min/1.73 m^2^), significant valvular heart disease and previous MI within 6 months. We also excluded patient data when flow cytometry quality standards could not be met.

*Measurements*

*Flow cytometry*

Monocyte subsets were analysed using a BD FACSCalibur flow cytometer (Becton Dickinson, Oxford, UK) as described previously [2, 3]. Monocyte subsets were defined in accordance with consensus guidance: Mon1 (CD14^++^CD16^-^), Mon2 (CD14^++^CD16^+^CCR2^+^) and Mon3 (CD14^+^CD16^++^CCR2^-^) [4]. Mouse anti-human monoclonal fluorochrome-conjugated antibodies (anti-CD16-Alexa Fluor 488, AbD Serotec, Oxford, UK; anti-CD14-PE, BD; anti-CD42a-PerCP, BD; and anti-CCR2-APC, R&D Systems, Abingdon, UK) were mixed with 50 µL of fresh ethylenediaminete-traacetic acid (EDTA) anticoagulated whole blood in TruCount tubes, BD containing a defined number of fluorescent count beads. We used isotype controls for protocol setup [2]. After incubation for 15 min, red blood cells were lysed using 450 µL of lysing solution (BD) for 15 min, diluted in 1.5 mL of PBS and immediately analysed. Absolute count of monocyte subsets was calculated following the manufacturer’s recommendations. This technique is robust and highly reproducible (coefficient of variation for absolute monocyte count is 1.9% and for surface markers is <5%) [2].

The phagocytic activity of monocyte subsets was measured using an established flow cytometry-based assay, pHrodo *Escherichia coli* BioParticles Phagocytosis kit for Flow Cytometry (Invitrogen, Carlsbad, CA, USA).[2] The assay utilises fluorescence of pHrodoTM *E.* *coli* BioParticlesTM-conjugates, inactivated, unopsonized *E. Coli* labelled with the fluorochrome with minimal fluorescence at neutral pH (e.g. in blood) which increases in acidic conditions (*i.e.* inside phagocytome). CD16-Alexa Fluor 488 (as above) and CD14-APC (BD) were used to define monocyte subsets. The phagocytic activity is reported as median fluorescent activity (MFI). The average laboratory coefficient of variation for the assay was 4.6%.

*Assessment of intracellular activation of nuclear factor κB (NFκB) pathway*

The cytoplasmic marker of activation of the NFκB pathway, IKKβ, was measured from fresh blood by flow cytometry as reported previously [2]. Fresh whole blood (100 µL) was incubated for 15 min with monoclonal mouse anti-human antibodies against CD16-Alexa Fluor 488 (AbDSerotec) and CD14-PerCp-Cy5.5 (BD), red blood cells were lysed using 2 mL of BD PharmLyseTM for 10 min and washed in staining buffer. The resulting pellet was resuspended in fixation/permeabilization solution (BD) for 20 min, centrifuged, kept in 2 mL of BD Perm/WashTM buffer for 10 min, centrifuged again and incubated for 30 min with monoclonal mouse antihuman APC-conjugated antibodies (LL-APC-XL conjugation kit; Innova Bio-sciences, Cambridge, UK) against IKKβ (Abcam, Cambridge, UK), washed and resuspended in 200 µL of 2% PBS/2% paraformaldehyde solution (PharmFix; BD) for immediate flow cytometric analysis.

*Echocardiography*

Transthoracic echocardiography was performed at 3 days and 6 months after STEMI and PCI, using Philips iE33 echocardiography machine with multifrequency phased-array transducer (1.5 MHz) following current recommendations across all sites [5]. LVEF measurements were made by an experienced technician or cardiologist in triplicate and averaged according to the recommendations of the American Society of Echocardiography [6]. LVEF at 6 month was used as echocardiographic outcome of cardiac function.

*Statistical analysis*

Continuous data are presented as mean ± standard deviation for normal data and as median and interquartile range [IQR] for non-normal data. A Wilcoxon Rank non-parametric test has been used to compare baseline characteristics between patients with MACE and without MACE, and to compare the dynamic changes between delta (post-pre) and baseline monocyte subsets. Univariate cox proportional hazard ratios (HR) were determined to assess the predictive value of monocyte subsets for survival from the study outcomes. Significant univariate predictors were fitted into multivariable models to determine their independent predictive value compared to the reference model (consisting of age, sex, maximal troponin T levels and estimated glomerular filtration rate, and history of diabetes and smoking as additional predictor variables given their recognized prognostic indication in STEMI). Kaplan-Meier curves were constructed to assess survival according to relative median change (post-pre). Similarly, linear regression was used to establish the predictive value of monocyte characteristics for 6-month LVEF. All statistical analyses were performed in R version 4.1.3 (PBC, Boston, MA, USA) using the following packages: survival [7] and survminer [8]. P-values <0.05 were considered statistically significant. For multivariate analyses, p-values <0.01 were considered statistically significant.

**References**

[1] Van de Werf, F, Bax, J, Betriu, A, et al., Management of acute myocardial infarction in patients presenting with persistent ST-segment elevation: the Task Force on the Management of ST-Segment Elevation Acute Myocardial Infarction of the European Society of Cardiology, Eur Heart J, 2008;29:2909-2945, <https://doi.org/10.1093/eurheartj/ehn416>

[2] Shantsila, E, Wrigley, B, Tapp, L, et al., Immunophenotypic characterization of human monocyte subsets: possible implications for cardiovascular disease pathophysiology, J Thromb Haemost, 2011;9:1056-1066, <https://doi.org/10.1111/j.1538-7836.2011.04244.x>

[3] Tapp, LD, Shantsila, E, Wrigley, BJ, et al., The CD14++CD16+ monocyte subset and monocyte-platelet interactions in patients with ST-elevation myocardial infarction, J Thromb Haemost, 2012;10:1231-1241, <https://doi.org/10.1111/j.1538-7836.2011.04603.x>

[4] Weber, C, Shantsila, E, Hristov, M, et al., Role and analysis of monocyte subsets in cardiovascular disease. Joint consensus document of the European Society of Cardiology (ESC) Working Groups "Atherosclerosis & Vascular Biology" and "Thrombosis", Thromb Haemost, 2016;116:626-637, <https://doi.org/10.1160/th16-02-0091>

[5] Lang, RM, Bierig, M, Devereux, RB, et al., Recommendations for chamber quantification: a report from the American Society of Echocardiography's Guidelines and Standards Committee and the Chamber Quantification Writing Group, developed in conjunction with the European Association of Echocardiography, a branch of the European Society of Cardiology, J Am Soc Echocardiogr, 2005;18:1440-1463, <https://doi.org/10.1016/j.echo.2005.10.005>

[6] Lang, RM, Bierig, M, Devereux, RB, et al., Recommendations for chamber quantification, Eur J Echocardiogr, 2006;7:79-108, <https://doi.org/10.1016/j.euje.2005.12.014>

[7] Therneau, TM, A Package for Survival Analysis in R, In, 2022.

[8] Alboukadel, K, Marcin, K, Przemyslaw, B, et al., survminer: Drawing Survival Curves using 'ggplot2', In, 2021.
