# Supplementary material for: Dynamic changes of monocytes subsets predict major adverse cardiovascular events and left ventricular function after STEMI
Source: Sci Rep. 2023 Jan 2;13:48. doi: 10.1038/s41598-022-26688-9 (PMC9807564; doi:10.1038/s41598-022-26688-9)
Supplement: Supplementary file 2 — Supplementary Information 2. [file 41598_2022_26688_MOESM2_ESM.docx]

**Online Resource 2.** Dynamic changes in monocyte characteristics.

|  | Baseline | 1 week | | 2 weeks | | 1 month | | |
| --- | --- | --- | --- | --- | --- | --- | --- | --- |
|  | Absolute values | Absolute values | Change | Absolute values | Change | Absolute values | Change | |
| *Count, cells/µL (n=42 for week 1, n=48 for week 2, n=62 for month 1)* | | | | | | | |  |
| Mon1 | 615 [487-841] | 641 [497-847] | -91 [(-304)-56.5]^†^ | 398 [325-486] | 10 [(-6)-31]^†^ | 487 [357-580] | -201 [(-401)-(-87)]^†^ | |
| Mon2 | 118 [62-228] | 35 [23-92] | -9 [(-38)-18]^†^ | 68 [32-98] | -45 [(-97)-(-6)]^†^ | 57 [23-100] | -41 [(-102)-(-10)]^†^ | |
| Mon3 | 54 [36-72] | 63 [45-89] | 10 [(-6)-31]^†^ | 43 [31-65] | -3 [(-19)-7]^†^ | 58 [38-75] | -8 [(-23)-15]^†^ | |
| *Phagocytic activity, MFI (n=4 for week 1, n=23 for week 2, n=25 for month 1)* | | | | | | | |  |
| Mon1 | 118 [100-145] | Not analysed^‡^ | Not analysed^‡^ | 129 [117-157] | 23 [(-4)-54]^†^ | 131 [110-142] | 12 [(-15)-38]^†^ | |
| Mon2 | 119 [92-144] | Not analysed^‡^ | Not analysed^‡^ | 143 [114-160] | 46 [7-61]^†^ | 120 [98-139] | 5 [(-25)-45]^†^ | |
| Mon3 | 42 [29-57] | Not analysed^‡^ | Not analysed^‡^ | 84 [63-112] | 39 [27-76] | 75 [63-112] | 31 [11-64] | |
| *Intracellular levels of inhibitory κB kinase β (IKKβ), MFI (n=34 for week 1, n=45 for week 2, n=54 for month 1)* | | | | | | | |  |
| Mon1 | 69 [55-86] | 66 [59-76] | -11 [(-19)-(-1)]^†^ | 61 [52-76] | -6 [(-21)-9] | 61 [48-73] | -11 [(-26)-2] ^†^ | |
| Mon2 | 67 [55-83] | 70 [66-85] | -16 [(-24)-(-1)]^†^ | 64 [50-79] | -5 [(-18)-10]^†^ | 64 [50-76] | -7 [(-31)-5]^†^ | |
| Mon3 | 70 [59-81] | 68 [56-79] | -10 [(-19)-4]^†^ | 68 [50-82] | -4 [(-16)-8]^†^ | 59 [52-73] | -12 [(-24)-0]^†^ | |
| Continuous variables are expressed as median [IQR]. Dynamic changes represent post value – pre value  MFI: median fluorescent intensity.  ^†^p<0.001vs baseline value; ^‡^Analyses were not performed due to insufficient number of samples. | | | | | | | |  |
