# Supplementary material for: Dynamic changes of monocytes subsets predict major adverse cardiovascular events and left ventricular function after STEMI
Source: Sci Rep. 2023 Jan 2;13:48. doi: 10.1038/s41598-022-26688-9 (PMC9807564; doi:10.1038/s41598-022-26688-9)
Supplement: Supplementary file 3 — Supplementary Information 3. [file 41598_2022_26688_MOESM3_ESM.docx]

**Online Resource 3.** Predictive value of changes of monocyte subsets for changes in phagocytic activity and Intracellular levels of inhibitory κB kinase β.

|  | **Univariate** |  |  |  | | **Multivariate** |  |  |
| --- | --- | --- | --- | --- | --- | --- | --- | --- |
|  | **B±SE** | ***β*** | **p-value** |  | | **B±SE** | ***β*** | **p-value** |
| ***Phagocytic activity*** | | | | | | | | |
| ***Changes at week 2 (n=23)*** | | | | | | ***(n=15)*** | | |
| Mon1 | 0.002±0.002 | 0.181 | 0.41 |  | | -0.003±0.011 | -0.132 | 0.77 |
| Mon2 | 0.003±0.002 | 0.246 | 0.26 |  | | 0.004±0.011 | 0.175 | 0.73 |
| Mon3 | 0.004±0.003 | 0.323 | 0.13 |  | | -0.001±0.007 | -0.060 | 0.90 |
| ***Changes at month 1 (n=25)*** | | | | | | ***(n=20)*** |  |  |
| Mon1 | 0.001±0.003 | 0.091 | 0.67 |  | | -0.003±0.004 | -0.235 | 0.42 |
| Mon2 | -0.007±0.004 | -0.346 | 0.09 |  | | -0.004±0.004 | -0.244 | 0.31 |
| Mon3 | -0.005±0.002 | -0.392 | 0.05 |  | | -0.003±0.003 | -0.319 | 0.24 |
| ***IKKβ*** | | | | | | | | |
| ***Changes at week 1 (n=35)*** | | | | | ***(n=31)*** | | | |
| Mon1 | 0.000±0.003 | 0.030 | 0.86 |  | | 0.000±0.003 | -0.008 | 0.97 |
| Mon2 | 0.001±0.002 | 0.097 | 0.58 |  | | 0.001±0.002 | 0.044 | 0.79 |
| Mon3 | -0.002±0.003 | -0.092 | 0.60 |  | | -0.002±0.004 | -0.094 | 0.59 |
| **Changes at week 2 *(n=46)*** | | | | | | ***(n=29)*** | | |
| Mon1 | 0.002±0.002 | 0.103 | 0.50 |  | | -0.001±0.003 | -0.088 | 0.68 |
| Mon2 | 0.002±0.003 | 0.105 | 0.49 |  | | -0.002±0.004 | -0.111 | 0.61 |
| Mon3 | -0.001±0.003 | -0.073 | 0.63 |  | | -0.002±0.005 | -0.107 | 0.63 |
| **Changes at month 1 *(n=55)*** | | | | | | ***(n=44)*** | | |
| Mon1 | 0.001±0.002 | 0.043 | 0.76 |  | | 0.003±0.002 | 0.171 | 0.18 |
| Mon2 | 0.002±0.002 | 0.152 | 0.27 |  | | 0.003±0.002 | 0.198 | 0.14 |
| Mon3 | 0.001±0.002 | 0.071 | 0.61 |  | | 0.004±0.003 | 0.212 | 0.11 |
| B: regression coefficient; SE: Standard error; *β*: adjusted regression coefficient; IKKβ:Intracellular levels of inhibitory κB kinase β  Multivariate analyses were performed using age, sex, maximal troponin T levels, estimated glomerular filtration rate, history of diabetes and smoking and monocyte subset counts. | | | | | | | | |
